# Transcriptome Sequencing and Metabolism Analysis Reveals the role of Cyanidin Metabolism in Dark-red Onion (*Allium cepa* L.) Bulbs

**DOI:** 10.1038/s41598-018-32472-5

**Published:** 2018-09-20

**Authors:** Chunsha Zhang, Xiaojie Li, Zongxiang Zhan, Linjiao Cao, Aisong Zeng, Guojun Chang, Yi Liang

**Affiliations:** 1 Beijing Vegetable Research Center, Beijing Academy of Agriculture and Forestry Sciences/Key Laboratory of Biology and Genetic Improvement of Horticultural Crops (North China), Beijing, 100097 China; 2 National Key Laboratory of Crop Genetic Improvement and College of Plant Science and Technology, Huazhong Agricultural University, Wuhan, 430070 China; 3 Horticulture College, Henan Agricultural University, Zhengzhou, 450002 China; 4 Key Laboratory for Horticultural Crop Genetic Improvement/Institute of Vegetable Crops, Jiangsu Academy of Agricultural Sciences, Nanjing, 210014 China; 5 Vegetable Research Institute, Jiuquan Academy of Agricultural Sciences, Jiuquan, 735000 China

## Abstract

Onion (*Allium cepa* L.) is an important bulbous vegetable crop that possesses important properties related to health as well as extraordinary colors. Naturally white onion bulbs were used in this study to reveal the complex metabolic mechanisms that underlie phenotypic traits, especially bulb pigmentation. Six libraries (three dark-red and three white) were constructed and analyzed to elucidate differences in cyanidin (Cy) metabolism between dark-red and white onion bulbs. Libraries were screened using RNA-sequencing (RNA-seq) to reveal the differentially expressed genes (DEGs) involved in anthocyanin biosynthesis at the transcriptional level. Comparison with the Kyoto Encyclopedia of Genes and Genomes (KEGG) database shows that a total of 27 unigenes participate in onion anthocyanin biosynthesis and 16 DEGs perform critical roles in flavonoid biosynthesis. Expression patterns of color-related flavonoid compounds associated with the onion anthocyanin biosynthesis pathway (ABP) show that flavonoid 3′,5′-hydroxylase (F3′5′H) and dihydroflavonol 4-reductase (DFR) genes play crucial roles in the biosynthesis of dark-red bulbs, the expression levels of flavonol synthase (FLS) and DFR genes may act to block blue pigmentation, and the loss of Cy from white onion bulbs might explain multibranching in the synthesis of this compound. Positive variation in the F3′5′H/F3′H ratio also affects onion bulb color diversity. The transcriptome presented here provides a basis for future onion molecular breeding based on variations in the diversity of ornamental plant pigmentation.

## Introduction

Onion (*Allium cepa* L.) is classified within the family Alliaceae and is an important and well-known vegetable used for cooking. These plants are an important source of nutrients and antioxidants in human diets, are known to have numerous health-related benefits, and are popular because they can mitigate the effects of diabetes, bronchial asthma, and cardiovascular disease^1–3^. Onion bulb color varieties range from white, to pink, reddish-purple, and dark-red; the appeal of these different varieties depends on their colors and the fact that pigmentation imparts resistance to onion smudge, Colletotrichum circinans (Berk.). Thus, as purely white bulbs are prone to infection^4^, onion pigments are important not just for their benefits to human health, but because of resultant disease-resistance during breeding selection for novel varieties. Therefore, understanding the molecular mechanism (s) that underlie color inheritance in onion bulbs is critically important.

Flavonoids are common plant secondary metabolites belonging to the class of phenylpropanoids class and occur in various modified forms. One of the best known flavonoid functions is in pigmentation; the colors of various vegetables, flowers, and fruits are the result of specific flavonoids known as anthocyanin compounds. Specific flavonoids, the anthocyanins, essentially contribute to the coloration of fruits and vegetables in many species. The dominant pigments in onion bulbs are cyanidin (Cy) and delphinidin glycosides^5–8^. Anthocyanins, one class of flavonoids that have a basic C6-C3-C6 structure, are responsible for the orange-to-blue colors seen in many color plants, are water-soluble, and are stored in plant vacuoles. The three main branches (Cy, Pg and Del) comprise the anthocyanin biosynthesis pathway (ABP) and determine the orange-red, deep red, and blue hues of onions; These differences in the number of hydroxyl groups present in B-rings such that a higher quantity results in a blueish color. Similarly, a concomitant increase in the number of methyl groups results in a redder pigmentation^9^. Although a number of published reports have addressed anthocyanin metabolism in ornamental flowers and plants with colored bulbs^10,11^, the genetic mechanisms involved in the loss of this dark-red pigment in white onion bulbs have not so far been addressed.

**Figure 1:**
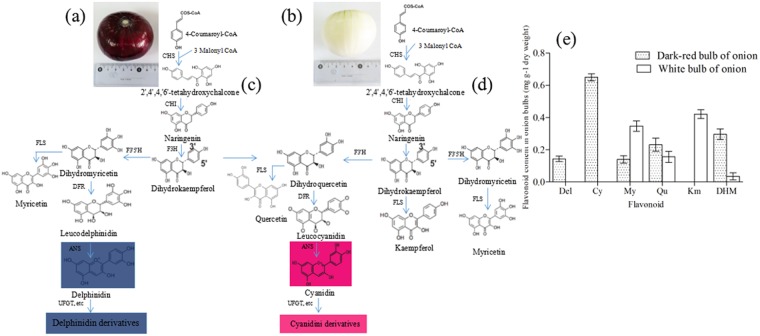
Diagram to show the putative anthocyanin metabolic process in dark-red and white onion bulbs. (**a**,**b**) Mature dark-red and white onion bulbs; (**c**) The likely anthocyanin metabolic process in dark-red onion bulbs; (**d**) The likely anthocyanin metabolic process in white onion bulbs; (**e**) Flavonoid composition obtained by HPLC from dark-red and white onion bulbs. CHS: chalcone synthase; CHI: chalcone isomerase; F3H: flavanone 3-hydroxylase; F3′5′H: Flavonoid 3′,5′-hydroxylase; F3′H: flavonoid 3′-hydroxylase; DFR: dihydroflavonol 4-reductase; DHM: dihydrokaempferol; FLS, flavonol synthase; ANS: Anthocyanidin synthase; UFGT: UDP-flavonoid glucosyltransferase.

The level of onion bulb pigmentation is closely-related to anthocyanin metabolism^2,12^, a process that takes place in the cytosol and involves various enzymes. For example, chalcone synthase (CHS), the first committed enzyme, catalyzes the chalcone 2′,4′,4,6′-tetahydroxychalcone (THC) that is produced by one molecule of 4-coumaroyl CoA and three molecules of malonyl CoA. THC is then isomerized to colorless naringenin by chalcone isomerase (CHI), naringenin is hydroxylated by flavanone 3-hydroxylase (F3H) to produce dihydrokaempferol, while flavonoid 3′,5′-hydroxylase (F3′5′H) and flavonoid 3′-hydroxylase (F3′H) catalyze the hydroxylation of dihydrokaempferol (DHM) to produce dihydromyricetin and dihydroquercetin, respectively. These flavonoids are essential for the production of Del and Cy, respectively; thus, dihydroflavonols are reduced to lecoanthocyanidins via the effects of dihydroflavonol 4-reductase (DFR) and are converted to quercetin, kaempferol, and myricetin by flavonol synthase (FLS). At the same time, the leucoanthocyanidin dioxygenase anthocyanidin synthase (ANS) catalyzes correspondingly-colored anthocyanidins. These compounds are subsequently converted to anthocyanins by the UDP-flavonoid glucosyltransferase (UFGT) as well as some glucosyl/acyl/methyl-transferases, and are then transported into the vacuole via three different pathways (Fig. 1)^9,13^.

The inheritance of onion bulb colors is a complex pattern. Previous studies have revealed the presence of five major genes that are responsible for bulb colors^14,15^. The first gene, *I*, is a color-inhibiting factor which is not completely dominant over *i*; thus, when this gene occurs in a homozygous dominant condition, it inhibits the expression of onion bublb color, and all onion bulbs will be white regardless of the genotypes of the other genes. The second gene, *C*, is a basic color factor that is completely dominant over *c* and is required for the production of all colors. This means that homozygous recessive (*cc*) plants will have white bulbs which are referred to as recessive white to distinguish them from their dominant white counterparts. The third factor, *G*, causes a golden yellow color when present in either homozygous or heterozygous condition. Nevertheless, recessive homozygous (*gg*) plants appear chartreuse-colored bulbs. The last two genes, *L* and *R*, play complementary roles and produce red pigments when present in either homozygous or heterozygous conditions. Previous research has shown that the DFR gene transcript only accumulate in red onions while inactivation of this enzyme precludes the anthocyanin produced in the yellow onions^15^. It is nevertheless worth discussing which colors will be produced in onion bulbs by low level DFR expression.

Previous reports on colored bulbous plants, including potato (*Solanum tuberosum* L.)^16^, carrot (*Daucus carota* L.)^17^, and radish (*Raphanus sativus* L.)^18^, have emphasized anthocyanin metabolism. These vacuolar pigments are known to have important human health benefits and functions in plant stress responses. As in other bulb plants, onion pigments are known to be important in anthocyanin metabolism. Thus, RNA sequencing (RNA-seq) was performed in this study to further elucidate the metabolic pathways involving these compounds in dark-red onion bulbs. The RNA-seq method is an easy-to-use and efficient tool for detecting novel differentially expressed genes (DEGs)^19,20^. The aims of this study were therefore to: (1) Identify DEGs involved in the anthocyanin pathway of onion bulb pigmentation, and; (2) Determine candidate genes targeting the loss of pigmentation in onion bulbs.

## Results and Discussion

### Major color compounds in onion bulbs

We compared the metabolic profiles of onion bulb skin samples to evaluate phenotypes that lack color and to quantify the compounds involved in pigmentation. The major anthocyanin and flavonols composition of the purified in dark-red and white onions were identified by UPLC-PDA-Triple-TOF-MS. The results of this study predictably show that dark-red onion bulbs contain the anthocyanin delphindin and cyanidin, the flavonoids myricetin, quercetin, kaempferol and dihydrokaempferol (Fig. 1e and Supplementary Table S1). The antheocyanin and flavonoids compounds cyanidin 3-glucoside and cyanidin 3-malonoylglucoside as well as delphinidin 3-diglglucoside, delphinidin 3-glucoside and delphinidin aglycon and that this pigment is absent from white bulbs (Table 1 and Supplementary Fig. S1). MS data were collected in TOF-MS scan-Information dependent acquisition (IDA)-Product ion scan mode. Thus, to further understand the absence of color, we compared the intermediate products of the ABP metabolic process to determine the key enzymes present in dark-red and white onion bulbs (Fig. 1c,d). In this context, the presence of myricetin, quercetin, and kaempferol in white bulbs is indicative of an interrupted downstream ABP gene such as DFR, ANS, or UFGT.

**Table 1:** Anthocyanins and flavonols identified from dark-red and white onion in this study.

Variety	No.	Retention Time (min)	Extraction Mass (Da)	Found At Mass (Da)	M+ (m/z)	Error (ppm)	Peak area	Peak assignment
Dark-red onion	Ant1	9.51	535.1088	535.1083	535	−0.73	23976666	Cyanidin 3-malonoylglucoside
	Ant2	20.52	449.1084	449.1081	449	−0.75	2290833	Cyanidin 3-glucoside
	Ant3	5.15	627.1561	627.1551	627	−0.79	49188	Delphinidin 3-diglucoside
	Ant4	27.63	465.1032	464.8732	465	−5.53	54303	Delphinidin 3-glucoside
	Ant5	34.54	356.2053	356.2033	303	−2.13	43804	Delphinidin aglycon
	Fla 1	11.02	310.3436	310.3406	289	−2.01	37803	Dihydrokaempferol
	Fla 2	26.31	348.9461	348.9441	319	−1.73	3065458	Myricetin
	Fla 3	36.82	364.5642	364.5636	303	−1.65	3136864	Quercetin
White onion	Fla 1	11.03	36.5111	36.4353	289	−2.78	3694	Dihydrokaempferol
	Fla 2	26.31	141.2461	141.673	319	2.13	11082	Myricetin
	Fla 3	36.82	33.2043	33.0111	303	−2.15	3132	Quercetin
	Fla 4	45.23	230.3412	230.3398	287	−1.93	23202	Kaempferol

### Total anthocyanin and flavonols content

The total anthocyanin contents determined by the pH differential method, were (35.87 ± 0.54) mg 100 g^−1^ in fresh dark-red onion and (1.42 ± 0.87) mg 100 g^−1^ in fresh white onion, respectively. In this study, the anthocyanin content in dark-red was much higher than in white onion. The total flavonoid contents were (142.21 ± 2.46) mg 100 g^−1^ and (0.03 ± 0.32) mg 100 g^−1^, in fresh dark-red and white onion, respectively. What’s more, the flavonoid content in white onion was far lower than that in dark-red onion (Table 2).

**Table 2:** Dark-red and white onion anthocyanin and flavonoids content (mean ± SE, *n* = 3).

Variety	Anthocyanin content (mg 100g-1 FW)	Flavonoids content (mg 100g-1 FW)
Dark-red onion	35.87 ± 0.54	142.21 ± 2.46
White onion	1.42 ± 0.87	0.03 ± 0.32

### RNA-seq and *de novo* assembly

Six libraries were constructed in this study (three dark-red and three white) in order to gain further insights into the molecular mechanisms of dark-red onion bulb pigmentation. These comprised a total of 48.02 Gb clean reads, no less than 6.86 Gb for each sample, corresponding to an average percentage of bases with sequencing error rates lower than 1% (Q30) out of more than 92.49% (Supplementary Table S2). These values indicate that RNA-seq data quality was sufficient for further analysis.

Subsequent assembly generated 31,859,070 contigs with a mean length of 41.16 basis pairs (bp), including a length encompassing 50% of all nucleotide sequences of the largest 43 bp unigene length (N50). The overall assembly integrity of sequences is high; detailed results are presented in Supplementary Table S3.

### Gene annotation and functional classification

**Figure 2:**
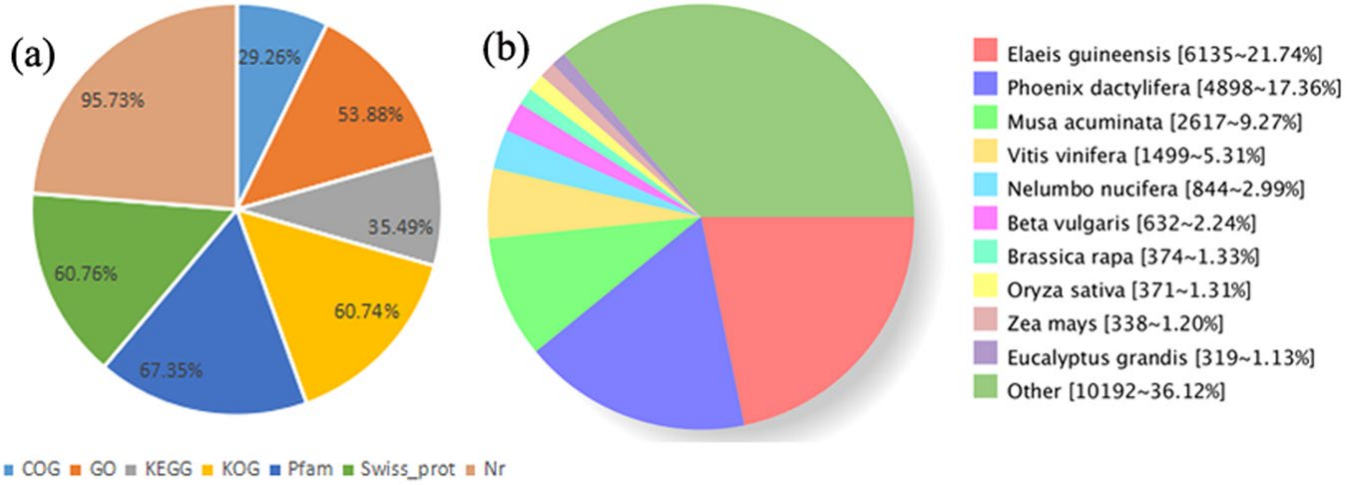
Unigene characteristics. (**a**) Different databases percentage; (**b**) Numbers and percentages of annotated unigenes matching major species found in the Nr database using BLAST.

The BLASTX parameters E-value < 1e^−5^ and HMMER < 1e^−10^ were applied in this study, leading to the generation of 29,491 (31.29%) unigenes which were then annotated via a series of versus databases (i.e., non-redundant protein (Nr), Swiss-Rort, clusters of orthologous groups (COG), eukaryotic orthologous groups (KOG), Pfam, gene ontology (GO), and Kyoto Encyclopedia of Genes and Genomes (KEEG)). Results show that 28,233 (95.73%) of annotated unigenes could be assigned to the Nr database, while the smallest proportion were assigned to the COG database (Fig. 2a). The distribution of major species is illustrated in Fig. 2b based on National Center for Biotechnology Information (NCBI) homology; data show that *Elaeis guineensis* (21.74%), *Phoenix dactylifera* (17.36%), and *Musa acuminata* (9.27%) all have high percentage homologies while the lowest value (1.13%) is seen in *Brassica rapa* and *Eucalyptus grandis*.

### DEGs related to the development of dark-red color

**Figure 3:**
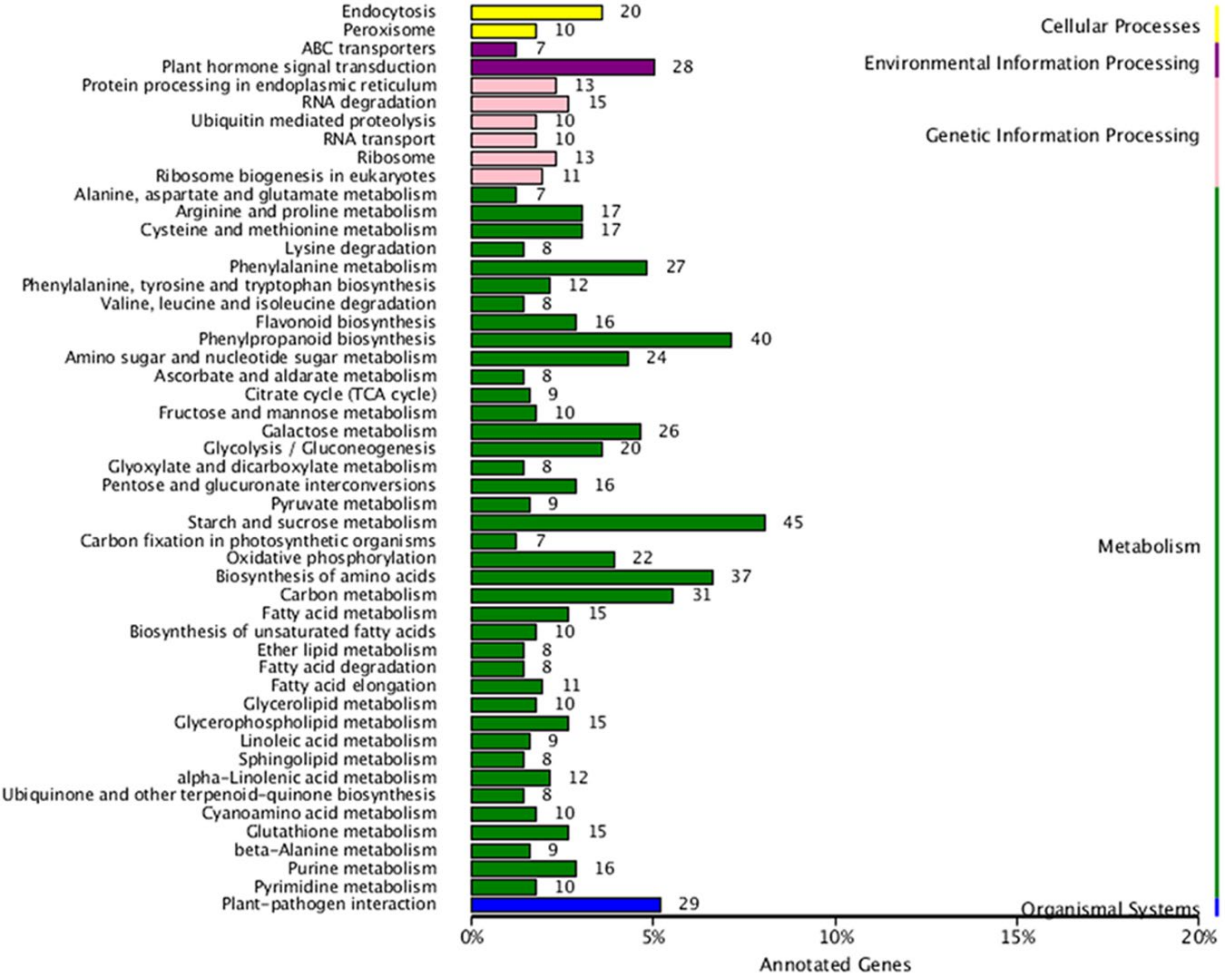
Comparison of KEGG enrichment DEG pathways between dark-red and white onion bulbs.

The unigenes obtained in this study were mapped against authoritative reference KEGG pathways to determine which are related to onion bulb colors. The results of this comparison revealed one secondary metabolic biosynthetic pathway (flavonoid biosynthesis) involved in color pigmentation; a total of 27 unigenes in total were assigned to this pathways (Table 3), while a total of 16 DEGs were shown to play key roles in flavonoid biosynthesis (Fig. 3 and Supplementary Table S4).

**Table 3:** Bulb pigmentation candidate genes in onion (*A*. *cepa* L.).

Function	Gene	Enzyme	KO id (EC no.)	No.All
Flavonoid biosynthesis	CHS	Chalcone synthase	K00660 (2.3.1.74)	2
	CHI	Chalcone isomerase	K01859 (5.5.1.6)	1
	F3H	Flavanone 3-hydroxylase	K00475 (1.14.11.9)	1
	F3′H	Flavanone 3′-hydroxylase	K05280 (1.14.13.21)	2
	F3′5′H	Flavanone 3′5′-hydroxylase	K13083 (1.14.13.88)	4
	DFR	Dihydroflavonol 4-reductase	K13082 (1.1.1.219)	1
	ANS	Anthocyanidin synthase	K05277 (1.14.11.19)	1
	UFGT	Anthocyanidin 3-O-glucosyltransferase	K12930 (2.4.1.115)	1
	C3M	Coumaroylquinate(coumaroylshikimate) 3′-monooxygenase	K09754 (1.14.13.36)	4
	CoA	Caffeoyl-CoA O-methyltransferase	K00588 (2.1.1.104)	3
	C4H	Cinnamate-4-hydroxylase	K00487 (1.14.13.11)	2
	SOHT	Shikimate O-hydroxycinnamoyltransferase	K13065 (2.3.1.133)	2
	FLS	Flavonol synthase	K05278 (1.14.11.23)	2
	FOMT	Flavonol 3-O-methyltransferase	K05279 (2.1.1.76)	1

### Comparisons of the transcriptional profiles of genes involved in anthocyanin metabolism in white and dark-red onion bulbs

**Figure 4:**
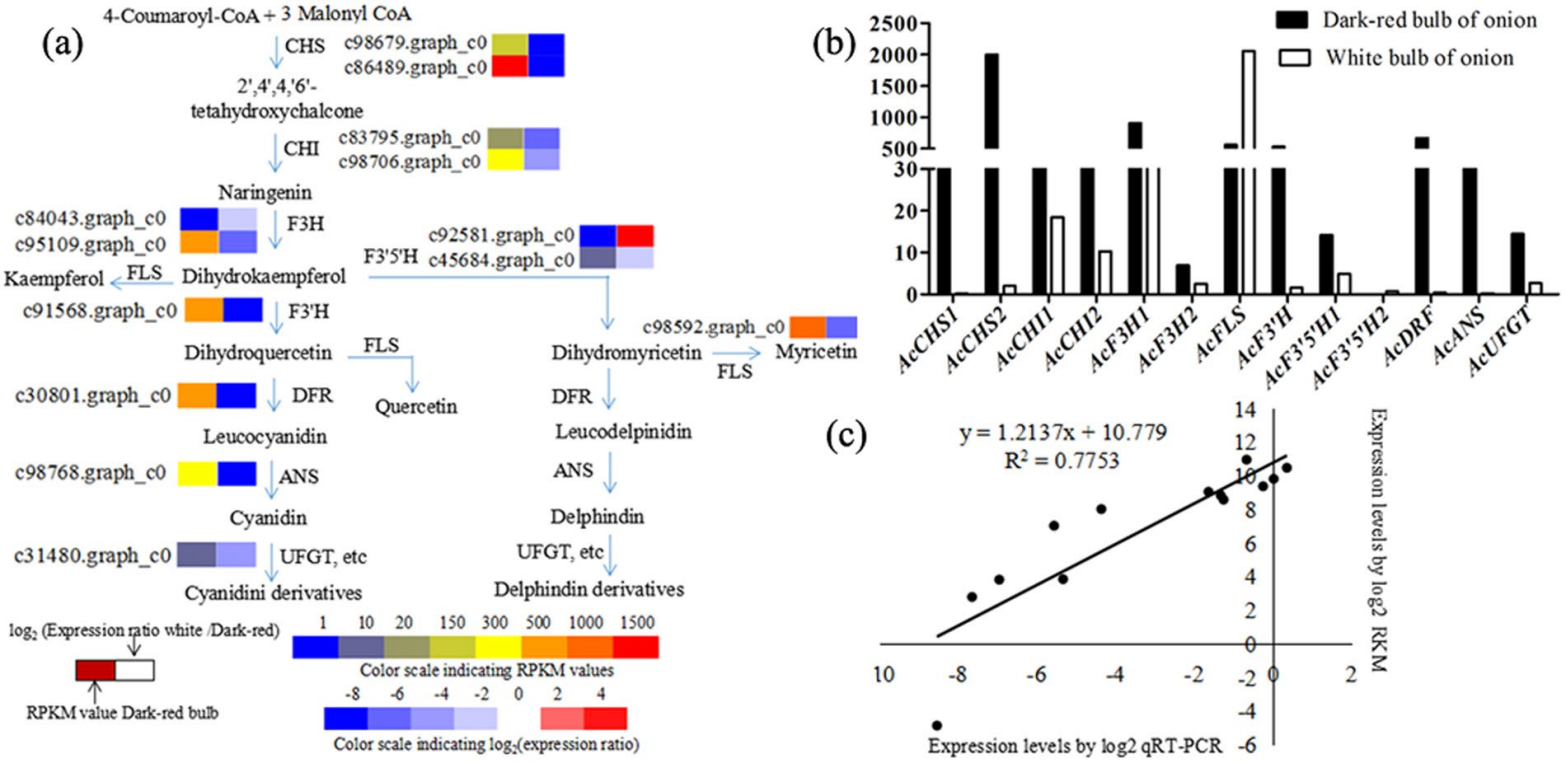
Physiological and metabolic data related to onion bulb pigmentation. (**a**) Detailed Cy and Del metabolic network showing subsets of nodes or metabolites that constitute this process. Enzyme and unigene names as well as expression patterns are listed next to each step, while expression patterns are summarized in two boxes; the PRKM values for dark-red onions are on the right, while the log2 expression ratio of white/dark-red are on the left; (**b**) Transcript accumulation of color-related genes involved in the anthocyanin metabolic process; (**c**) Gene expression correlation analysis for color-related genes in dark-red onion based on qRT-PCR and RNA-seq. Abbreviations as in Fig. 1.

Previous studies on plant bulbs and flowers have shown that loss of color from dark-red to white is the result of major deficiencies in the anthocyanins (Cy and Del)^8,11,17^. This is also the case in onion bulbs; a color transition from dark-red to white is due to an ABP blockage preventing reactions that would normally lead to the anthocyanins Cy and Del. We therefore compared the transcriptomes of dark-red and white onion bulbs and their associated RNA-seq annotations to determine the transcripts involved in dark-red color metabolism. The 13 critical DEGs implicated in anthocyanin metabolism were then subjected to qRT-PCR analysis using the primers designed in this study in order to verify our RNA-seq results,. These qRT-PCR results reveal that the expression levels of selected DEGs expression levels were generally consistent with transcriptome sequencing data. The transcriptome sequencing data reported in this paper are therefore credible and are further corroborated by a high correlation coefficient (R2 = 0.775) (Fig. 4c). These comparisons reveal significant changes in the expression levels of 13 key uni-transcripts (Fig. 4a,b); while most key genes were expressed in anthocyanin biosynthesis, it is noteworthy that the level of the F3′5′H1 in dark-red bulbs was more than three times that seen in white one (14.16 versus 4.89), while the expression of the F3′5′H2 was far below in the dark-red onion (0.03 versus 0.75) (Fig. 4b). Data also show that the expression levels of DFR, ANS, and UFGT in the dark-red bulbs were up to 30 times higher than in white bulbs counterparts; this result demonstrate that F3′5′H and DFR genes play an important role in anthocyanin biosynthesis in dark-red onion bulbs and also provide an explanation for the absence of kaempferol in white bulbs (Fig. 1e).

### Candidates which are responsible for the loss of Del and Cy in white onion bulbs

**Figure 5:**
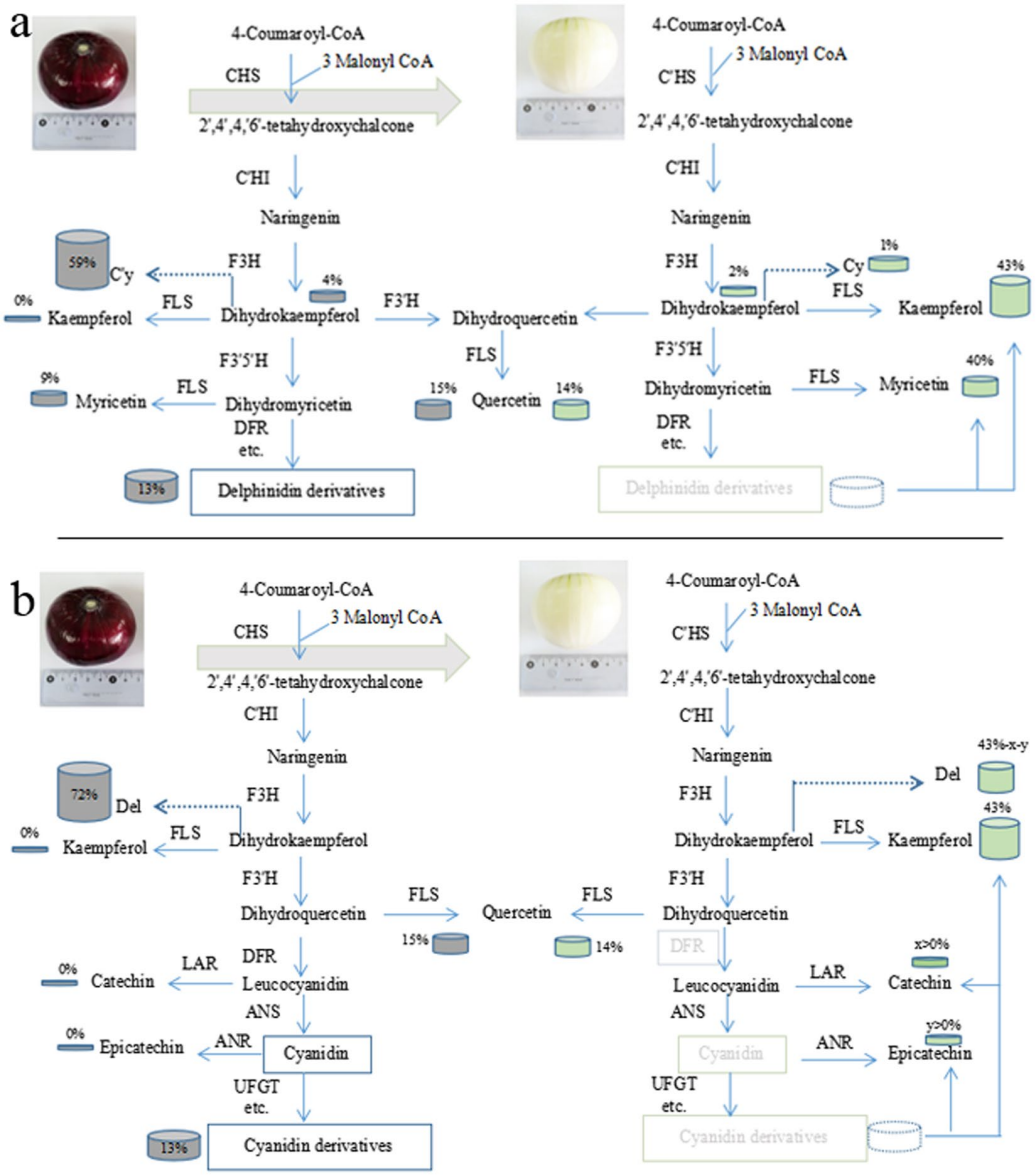
Ideal model for Del and Cy elimination from white onion bulbs. (**a**) Loss of Del from white onion bulbs. In cases when DFR is suppressed, substrates used for Del synthesis become available for synthesis of Kaempferol and myricetin. (**b**) Ideal model for the loss of Cy from white onion bulbs. The fluxe due to Cy metabolism is limited in this case, and downstream reactions promote the turnover and degradation of this pigment in the white onion bulbs. The global output from the minimal anthocyanin subnetwork in onion was considered to be 100% and defined the relative content on each product. The dark grey and light green boxes on this figure denote the relative content of genes or compounds in the dark-red and white onion bulbs, respectively. Abbreviations: LAR: leucoanthocyanidin reductase; ANR: anthocyandin reductase. All other abbreviations as in Fig. 1.

Although anthocyanidin metabolism in both white and dark-red onion bulbs involves the same enzymes prior to Del- and Cy-related reactions (Fig. 1c,d), the catalysis of subsequent specific reactions remains studied. It is generally acknowledged that CHS is the first catalyzing enzyme to produce the intermediate chalcones that are then processed in later metabolism^21^; thus, if this CHS enzyme is restricted, then both the production of anthocyanins and, subsequently, flavonoids will be limited and there will be no synergetic effect on the dark-red and white onion bulbs^22,23^. Previous studies have shown that F3′5′H plays an important role in the ABP pathway and is a prerequisite for the formation of Del (violet-to-blue) anthocyanins^24^; this implies that if the metabolism pathways for the minimal Del path of F3′5′H is cut, myricetin-related flavonols may not be determined in this study. However, as myricetin was detected in white onion bulbs anthocyanin at levels more than twice those in the dark-red onion (Fig. 1e), this explanation for the absence of Del is unsatisfactory. We therefore also addressed the question of whether, or not, the DFR gene which is crucial for anthocyanin formation can explain the absence of Del in the white onions; as this gene leads to colorless leucoanthocyanidins and no products of Del biosynthesis were found subsequent to dihydromyricetin in the white bulbs (Fig. 5a); DFR may be a candidate gene for this blocking process. At the same time, dihydroflavonols substrates represent branch points for the production of colourless flavonols (Km, My and Qu) through FLS; indeed, the expression levels of this gene are gradually up-regulated due to competing substrate (Fig. 4b). These results of this study therefore also reveal that both *FLS* and *DFR* genes catalyze the formation of dihydromyricetin and block Del synthesis, which is consistent with the result of earlier work^25^.

Metabolism and expression patterns of dark-red and white onion bulbs were compared to determine candidates for the loss of Cy accumulation. In this context, both the enzymes FLS (c98592.graph_c0) and DFR (c30801.graph_c0) are likely to limit Cy accumulation, in agreement with the earlier work^11^; up-regulation of *FLS* occurs when substrates are available to catelyze with Km in white onion bulbs (Fig. 1e), while the down-regulation of DFR could be unable to completely block the Cy process. A number of recent studies have discussed the use of new approaches for detecting the loss of pigmentation phenotypes via blocking points in anthocyanin biosynthesis^26,27^; one report noted that when the anthocyandin reductase (ANR) gene in apple was introduced to tobacco, this limited expression of the *DFR* gene in apple blossoms. As a similar process might also explain the absence of anthocyanin^28^, we hypothesize that this might provide an ideal model for Cy elimination from white onion bulbs (Fig. 5b); even though, red Cy (which occurs in either very low concentrations or is present for only a very short time) is also determined via this mechanism. This result indicates that a complex metabolic mechanism underlies accumulation of this compound in the ABP that might be explained by a variety of factors (e.g., light, UV-A)^29^. It is clear that leucyanidin is formed before Cy and that this catalyzes two products (colourless catechin and red Cy) via leucocyanidin reductase (LAR) and ANS, respectively; this also means that Cy might be reduced to colorless epicatechin by ANR and so a stable form of this pigment might not exist. Ideally, catechin and epicatechin products should be detectable in white onion bulbs and absent from dark-red form, but further trials will be required to explore this transition in tuber plants. The loss of Cy from white onion bulbs might also provide an explanation multibranching of the Cy synthesis pathway.

### The enzymes F3′5′H and F3′H play important roles in the accumulation of Del and Cy

Results show that the enzymes F3′5′H and F3′H both control crucial steps in the accumulation of Del and Cy anthocyanins and lead to significant changes in expression levels (Figs 1 and 4b). Previous work on a range of flower colors and plant bulbs has shown that the ratio between the expression of these genes controls the proportion and composition of flavonoids. In blue grape berries (*Vitis vinifera*), for example, a higher ratio of F3′5′H/F3′H leads to the production of more Del derivatives than in red or white cultivars^25,30^, while different ratios of these enzymes in *Senecio cruentus* determines Del and Cy accumulation and is the reason for their range of red and blue color varieties^31^. Similarly, roses (*Roses hybrida*) and carnations lack violet and blue color varieties because they do not produce Del, perhaps due to the loss of the F3′5′H gene during evolution. The high-performance liquid chromatography (HPLC) analysis presented here reveals a higher Cy content compared to Del (Fig. 1e) in dark-red onion bulbs and indicates that the F3′5′H/F3′H ratio is lower than that of F3′H/F3′5′H. This might indicated that both the genes *F3*′5′*H* and *F3*′*H* from onion (*A*. *cepa* L.) do not have a synergetic effect in regulating pigmentation.

## Material and Methods

### Plant material

The dark-red onion (*A*. *cepa* L.) cultivar ‘Xiu Qiu’ (fourth generation inbred lines from the dark-red ‘Shanxi’ variety) and the white onion cultivar ‘Ring Master’(fourth generation inbred lines from the white ‘Xinjiang’ variety) were used in this study. We performed a series of genetic tests to confirm whether white onions were dominant or recessive; as the F_1_ color of (‘Xiu Qiu’ × ‘Ring master’) was pink, the genotype of the white parent was *i/i*, and the phenotypes of the F_2_ segregating population were white, yellow, pink, and dark-red (Supplementary Fig. S2).

Fresh, undamaged 7 cm diameter samples weighing between 280 g and 320 g were collected on the 40^th^ day after swelling from a test field at the Beijing Academy of Agriculture and Forestry Vegetable Research Center (Fig. 1a,b). Samples were washed, excess water was absorbed with blotting paper, and lengths between 1 cm and 2 cm from the top and the bottom of each were excised with a scalpel. Outer onion bulb layers were then divided into three parts for experiments; all materials were immediately frozen in liquid nitrogen (N) and stored at −80 °C prior to RNA extraction and flavonoid analyses.

### Extraction of athocyanins and flavonoids

The extraction method used in this study follows previously reported protocols^25,32^ with slight modifications. Onion bulb peels frozen with liquid N were ground into fine powder and 50 mg subsamples were placed into 1.5 ml EP tubes. Extraction was then performed using 1 ml methanol containing 1% formic acid (v/v) before samples were sonicated in an ultrasonic bath at 4 °C for 24 hours. Samples were then centrifuged at 13,000 rpm for 10 minutes, supernatants were extracted into fresh tubes, and the whole protocol was repeated. Before the analysis, the extract was fltered by using 0.22 µm reinforced nylon membrane flters. Deionized water containing 0.01% hydrochloric acid were added up to 25 mL after the remain supernatants were combined and evaporated under vacuum to dryness at 35 °C to analyze the detailed anthocyanin and flavonoids composition and their antioxidant activities. A 3 µl supernatant volume from each sample was then injected for UPLC-MS analysis.

### UPLC-PDA-Triple-TOF-MS analysis

Bulbs of differently-colored onions were subject to anthocyanin analysis using Shimadzu UHPLC LC-30A system (Shimadzu, Japan) coupled to an AB SCIEX 6600 Triple-TOF-MS (AB SCIEX, USA). Chromatographic separations were performed on an Acquity UPLC™ BEH C_18_ column (2.1 mm × 100 mm, i.d., 1.7 µm) (Waters Corp., MA, USA) at 40 °C Chromatographic separations were performed on an Acquity UPLC™ BEH C_18_ column (2.1 mm × 100 mm, i.d., 1.7 µm) (Waters Corp., MA, USA) at 40 °C. The solvent system consisted of water with 0.1% formic acid (mobile phase A) and acetonitrile with 0.1% formic acid (mobile phase B). The column was eluted with a linear gradient of 0–28% B over 0–22 min, 28–40% B over 22–22.5 min, 40–100% B over 22.5–23 min, the composition was held at 100% B for 2 min then returned to 0% B and re-equilibrated for an additional 3 min before injection of the next sample. The flow rate of mobile phase was 0.3 mL/min. PDA detector was set at 200~800 nm.

Mass spectrometry analysis was performed using an electrospray ionization source (ESI) in positive ion mode. The source parameters were set as follow: source temperature of 550 °C, ion spray voltage of 5.5 kV, declustering potential of 80 V, atomization gas pressure (GS1) of 0.34 MPa, air curtain gas (CUR) of 0.24 MPa and auxiliary air pressure (GS2) of 0.34 MPa. Nitrogen was used in all cases.

Anthocyanins (cyanidin 3-glucoside, cyanidin 3-malonylglucoside, delphinidin 3-diglycoside delphinidin 3-glucoside and delphinidin aglycon) and flavonols (quercetin, kaempferol, myricetin, and dihydromyricetin) standards were purchased from Sigma-Aldrich China (Shanghai); mean values and standard deviations from three biological replicates are reported in this study, expressed in milligrams per gram dry weight. Quantification of single compounds was achieved by peak area using corresponding standard samples^33,34^.

### Total anthocyanins and flavonols content measurement

Total anthocyanin content in dark-red and white onion was measured by using the PH differential spectrophotometric method with some modifications^35^. Taking dark-red and white onion bulb 1.0 g, respectively, after grinding fully and adding precooling 10 ml 0.005% hydrochloric acid-methanol solution, storing at 4 °C for 12 h avoiding light and then collecting supernatant. The remaining residue was treated in the same way, combining with these supernatants, respectitvely. Taking the solution (1 ml) was dissolved with 0.025 mol L^−1^ potassium chloride buffer (PH = 1.0) and 0.4 mol L^−1^ sodium acetate buffer, respectivelly, and metered volume (25 ml). The absorbance was measured at 525 nm and 700 nm. Absorbance (*A*) of the diluted samples were then calculated as follows: anthocyanins content (mg L^−1^) = [(OD_525_ − OD_700_) PH1.0 − (OD_525_ − OD_700_) PH4.5] × 449.2 × 1000/26900 × DF, among of them, 449.2 is the relative molecular mass of cyanidin-3-glucoside; 26,900 is the molar absorptivity; DF is the dilution factor; 1000 is the factor to convert g to mg.

The total flavonols content in dark-red and white onion was measured by spectrophotometric method^36^. Rutin was used as a standard compound. Sample spectral absorbance measurements were read at 510 nm. The flavonols were calculated from the calibration curve: R (mg mL^−1^) = 0.1565OD_750_ – 0.0011 (R^2^ = 0.9925) and flavonols content (mg L^−1^) = R × DF/m. Among of them, DF is 2,500, m is the quality of the onion samples.

### RNA extraction and RNA-seq library construction

Total RNA was isolated using the TRIzol reagent (Invitrogen, Carlsbad, CA, USA) following the manufacturer’s instructions but incorporating a few modifications including precipitation with isovolumetric isopropanol and a high-salt solution (1.2 mol/L sodium chloride and 0.8 mol/L sodium citrate) rather than isopropanol precipitation when the supernatant fluid was removed. RNA concentration was initially characterized on a 1% agarose gel, and then examined using a NanoDrop 2100 spectrophotometer (Thermo Fisher Scientific, USA) to ensure RNA quality. Integrity RNA numbers were assessed using an Agilent 2100 system (Agilent Technologies, CA, USA) and were between 8.9 and 10.0 in all cases. Library construction and RNA-seq analyses were completed by the Biomarker Biotechnology Corporation (Beijing, China) utilizing RNA from each sample.

Two complementary DNA (cDNA) libraries were generated and sequenced in this study using an Illumina HiSeq. 4000 system. Messenger RNA was enriched using oligo(dT)-attached magnetic beads and sequences were randomly broken into short fragments via the addition of a fragmentation buffer. These short fragments were then used as templates for the synthesis of first-and second-strand cDNA using random hexamers and the addition of buffer, dNTPs, RHase H, and DNA polymerase I, respectively. Fragments of cDNA were then purified, subjected to end-repair, the addition of poly (A) tailing and ligation sequencing adapters, and size-selected using AMPure XP beads. Suitable fragments were then placed on 2% agarose gel for use as Polymerase Chain Reaction (PCR) templates for the amplification of cDNA libraries.

### *De novo* transcriptome assembly and functional annotation

Raw pair-end (PE) 150 bp reads were initially filtered prior to assembly via the removal of adaptor and low quality reads as well as unknown nucleotides. The remaining high quality clean reads were then utilized for *de novo* transcriptome assembly using a Trinity platform (http://trinityrnaseq.sourceforge.net/) with the parameters ‘K-mer = 25’ and ‘group pairs distance = 300’ as well as other defaults and in the absence of a reference genome^37^. Thus, based on overlap regions, short reads were initially assembled into longer contigs which were then clustered to form components; the different constituent contigs of each were then used to build a De Bruijn diagram which was untangled using real reads to obtain transcriptional sequences that were clustered to obtain uni-transcripts using the TGI software clustering tool^38^.

The software BLAST (E-value ≤ le^−5^)^39^ was then used to compare resultant uni-transcript sequences against the non-redundant protein (Nr) (ftp://ftp.ncbi.nih.gov/blast/db/), Swiss-Prot (http://www.uniprot.org/), GO (http://www.geneontology.org/), COG (http://www.ncbi.nlm.nih.gov/COG/), KOG^40^, and KEGG (http://www.genome.jp/kegg/) databases. The software KOBAS2.0^41^ was then used to determine KEGG unigene orthology results; after predicting the amino acid sequence of each unigene, we then used the software HMMER^42^ to make comparisons with the Pfam database^43^ to obtain unigene annotation information.

### Differential expression analysis

The software Bowtie^44^ was used in this analysis to compare alignment results for the reads of each sample (i.e., three biological replicates in each case) versus the unigene library associated with RNA-seq utilizing expectation maximization (RSEM)^45^ to estimate expression level in each case. Thus, the expression abundance unigene differences among samples were represented by values of fragments per kilobase of transcript per million mapped reads (FPKM), and the software DESeq.^46^ was used to screen DEGs via pairwise comparisons. The universally recognized and effective Benjamini-Hochberg method was utilized to determine a significant P-value for the original hypothesis being tested in each case; corrected P-values incorporate false discovery rate (FDR) when screening DEG levels such that when the FDR was less than 0.001 and the log_2_ fold change (FC) was either less than −2 or greater than 2 in terms of FPKM between two libraries. FC values therefore denote the expression ratio between two samples.

### Gene validation and expression analysis

Real-time-quantitative PCR (qRT-PCT) was performed using a Roche Light Cycler 480 machine (Bio-Rad, USA) incorporating a 96 real-time system in order to validate RNA-seq results and the roles of key enzymes related to in the anthocyanin biosynthetic pathway. SYBR^®^ Premix Ex Taq^TM^ (Tli RNaseH Plus) (TaKaRa, Dalian, China) was used for all PCR reactions, and primers were designed using the software Primer Premier 5.0 (Supplementary Table S5). Synthesis of cDNA and qRT-PCR were performed using previously described methods^47^; three technical qRT-PCT replicates of each sample as well as two biological replicates were performed to ensure reliability, and the inference gene β-*actin*^48^ was used to normalize gene expression levels.

## Electronic supplementary material

Supplementary Information
