# Supplementary material for: Transcriptome Sequencing and Metabolism Analysis Reveals the role of Cyanidin Metabolism in Dark-red Onion (Allium cepa L.) Bulbs
Source: Sci Rep. 2018 Sep 20;8:14109. doi: 10.1038/s41598-018-32472-5 (PMC6148220; doi:10.1038/s41598-018-32472-5)
Supplement: Supplementary file 1 — Supplementary Information [file 41598_2018_32472_MOESM1_ESM.pdf]

Transcriptome Sequencing and Metabolism Analysis Reveals the role of Cyanidin Metabolism in Dark-red Onion (*Allium cepa* L.) Bulb

Chunsha Zhang[1], Xiaojie Li[1], Zongxiang Zhan[2], Linjiao Cao[3], Aisong Zeng[4], Guojun Chang[5] and Yi Liang[1]

[1]Beijing Vegetable Research Center, Beijing Academy of Agriculture and Forestry Sciences/ Key Laboratory of Biology and Genetic Improvement of Horticultural Crops (North China), Beijing100097, China

[2]National Key Laboratory of Crop Genetic Improvement and College of Plant Science and Technology, Huazhong Agricultural University, Wuhan430070, China

[3]Horticulture College， Henan Agricultural University, Zhengzhou 450002, China

[4]Key Laboratory for Horticultural Crop Genetic Improvement/Institute of Vegetable Crops, Jiangsu Academy of Agricultural Sciences, Nanjing210014, China

[5]Vegetable Research Institue , Jiuquan Academy of Agricultural Sciences, Jiuquan 735000, China

Correspondence and requests for materials should be addressed to Y. L. (liangyi@nercv.org)

Supplementary Figure S1. HPLC chromatograms of the dark-red and white bulb of onion.(A) The chromatogram of flavonoids in the dark-red and white bulb of onion. Dihydrokaempferol (DHM) ; Myricetin (My) ; Quercetin (Qu); kaempferol (Km) (B) The chromatogram of anthocyanins in the dark-red and white bulb of onion. 1, Delphinidin 3-diglycoside 2, cyanidin 3-malonoylucoside; 3, cyanidin 3-glucoside; 4, delphinidin 3-glucoside; 5, delphinidin aglycon. The black, pink and blue line represents dark-red, white bulb of onion and starndard of sample, respectively.

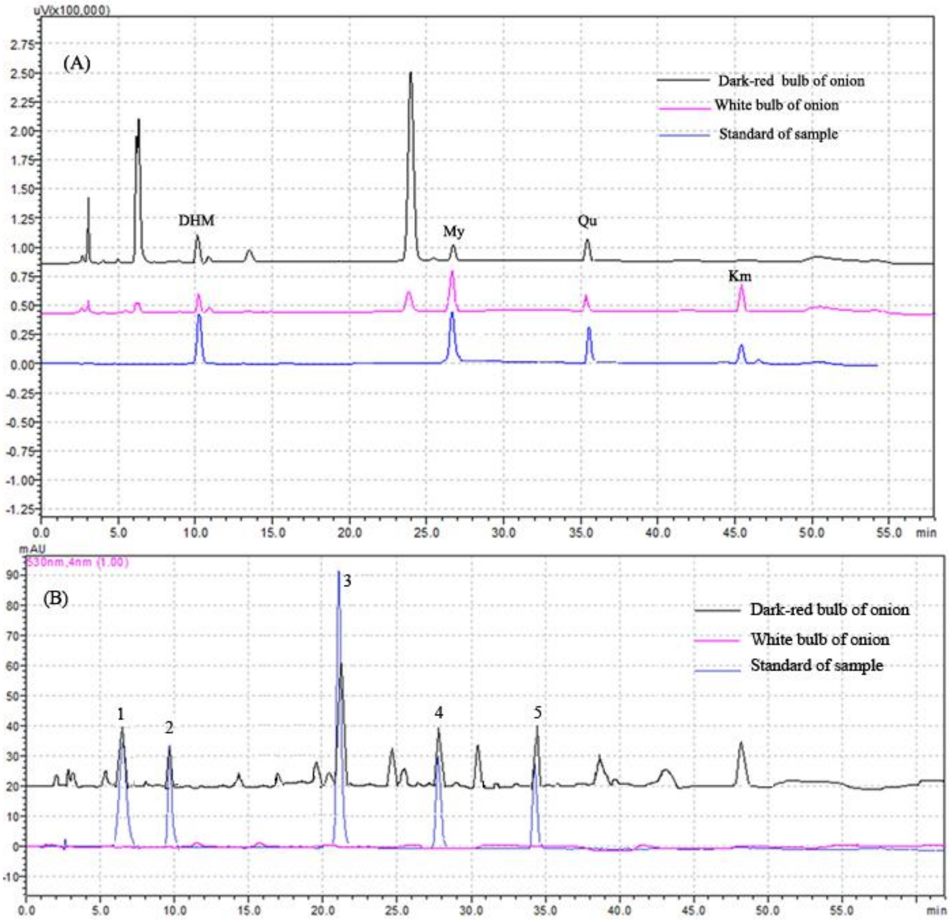

Supplementary Table S1 Two different color onion anthocyanin and flavonoids content (mean±SD, n=3)

	Standard	Dark-red onion	White onion
Anthocyanins(mg g-1)	Delphindin	0.143±0.017	ND
	Cyanidin	0.649±0.022	ND
Flavonols (mg g-1)	Myricetin	0.140±0.023	0.347±0.032
	Quercetin	0.231±0.04	0.156±0.034
	Kaempferol	ND	0.421±0.028
	Dihydrokaempferol	0.296±0.033	0.035±0.022

ND means not detected this compounds in the onion.

Supplementary Table S2 Sequencing data of each sample

Sample	Read Number	Base Number	GC Content	%≥Q30
W1	25,465,094	7,574,809,134	43.29%	92.49%
W2	32,170,124	9,561,335,218	43.39%	92.97%
W3	27,383,572	8,131,928,180	43.39%	92.82%
P1	23,822,626	7,087,829,958	43.80%	92.65%
P2	23,045,922	6,849,929,194	43.82%	92.76%
P3	29,620,569	8,816,608,584	43.69%	92.74%

Supplementary Table S3 Length distribution of contigs, transcripts, and unigenes in bulb of the onion (*Aillum cepa* L.) transcriptome

Length Range	Contig	Transcript	Unigene
200-300	31,716,169(99.55%)*	21,254(12.80%)	16,872(18.01%)
300-500	64,551(0.20%)	33,758(20.34%)	22,771(24.31%)
500-1000	46,263(0.15%)	49,697(29.94%)	27,174(29.01%)
1000-2000	23,026(0.07%)	39,490(23.79%)	17,782(18.99%)
2000+	9,061(0.03%)	21,795(13.13%)	9,060(9.67%)
Total Number	31,859,070	165,994	93,659
Total Length	1,311,237,265	176,445,502	84,628,953
N50 Length	43	1,567	1,347
Mean Length	41.16	1062.96	903.59

Note: * represent the number and percentage of the contig, transcript and unigene

Supplementary Table S4 Key DEGs of the flavonoid synthesis in onion (*A.cepa* L.) purple bulbs

Genes	Annotation
c30746.graph_c0	caffeoyl-CoA O-methyltransferase
c30801.graph_c0	dihydroflavonol 4-reductase
c37537.graph_c0	caffeoyl-CoA O-methyltransferase
c37537.graph_c1	caffeoyl-CoA 3-O-methyltransferase
c37991.graph_c0	cinammate 4-hydroxylase
c45684.graph_c0	flavonoid 3',5'-hydroxylase
c83795.graph_c0	Chalcone isomerase
c84043.graph_c0	flavanone 3-hydroxylase
c86129.graph_c0	shikimate O-hydroxycinnamoyltransferase
c86489.graph_c0	chalcone synthase
c91568.graph_c0	flavonoid 3'-hydroxylase
c92581.graph_c0	flavonoid 3',5'-hydroxylase
c98592.graph_c0	flavonol synthase
c98768.graph_c0	anthocyanidin synthase
c98910.graph_c0	shikimate O-hydroxycinnamoyltransferase
c99191.graph_c0	p-coumarate 3-hydroxylase

Supplementary Figure S2. Pedigree of a population originating from a cross between Dark-red and white onions ('Xiu Qiu'× 'Ring Master').

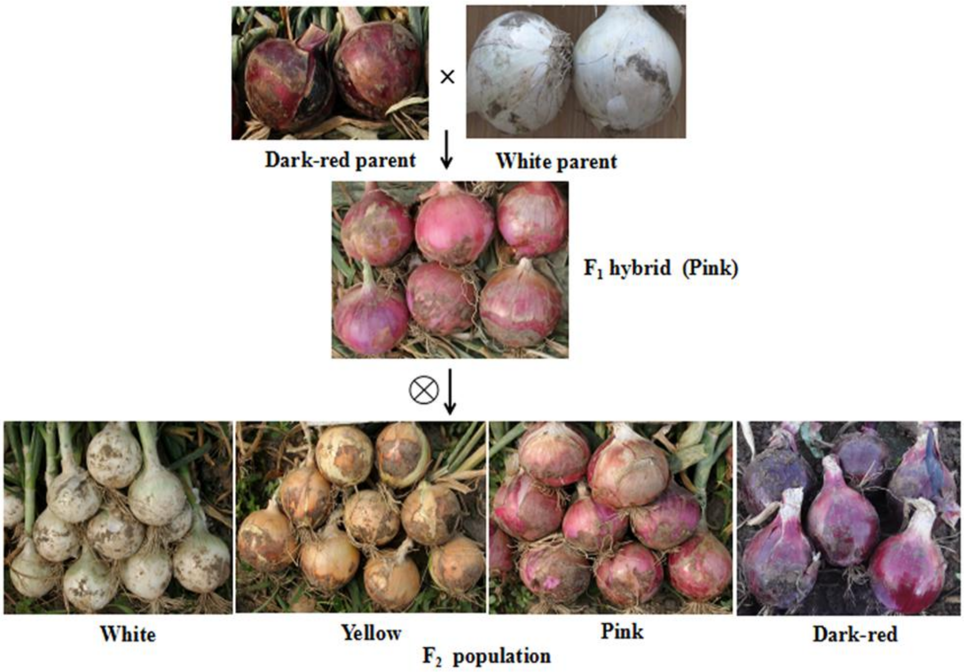

Supplementary Table S5 The primers provided for quantitative real-time PCR

Gene/Unigene	Forward	Reverse
c31408	TTGAAGGCGGTAATGGAG	CGAAAACAACCCGAACAC
c83795	AATGATTCTCCCTCTAACGG	TTCTCAATGAGTACGCCTTC
c86489	CGGCAAGACATCGTGGTT	ATCGCTCCTTCCGAATCC
c98679	CAACGACTCAAACAGCAAA	CGACTGGAGAAGGGATGG
c98768	ATTCCGATAACCGACCTC	CCAGTTTACTTCCATACCCT
c95109	CACATCCCAGACGCACTA	TCCAACTACAGGAACATAAGC
c91568	GTCCTTTATTTGGTCTACGG	GCCAGGCTCATTATTTCG
c92581	TCATCCGCTTCCACTTTC	ACATCACCAACTCCGACAG
c45684	CAGACCTACCAAATCTCCCT	CAACGCATACTCTTCTACCAG
c98592	ACGCTAACGGCAAACCTG	GCCTTCTTCCCTTCCAAA
c84043	AACTCCATTTCTACCCACA	ATTTCATCGCTAAACACG
c98706	TCCTTGAACATCTGAGCA	ACCCTTCTTGAAATACGG
c30801	ATGTTCAAGCAACTCATAAGGC	TAAAGCGGAAGGGCGATA
